# Interior Color and Psychological Functioning in a University Residence Hall

**DOI:** 10.3389/fpsyg.2018.01580

**Published:** 2018-08-28

**Authors:** Marco Costa, Sergio Frumento, Mattia Nese, Iacopo Predieri

**Affiliations:** ^1^Department of Psychology, University of Bologna, Bologna, Italy; ^2^Department of Surgery, Medical, Molecular, and Critical Area Pathology, University of Pisa, Pisa, Italy

**Keywords:** color, chromatic preference, lightness preference, architecture, interior design

## Abstract

The research exploited a unique architectural setting of a university residence hall composed by six separate buildings that matched for every architectural detail and differed only for the interior color (violet, blue, green, yellow, orange, and red). Four hundred and forty-three students living in the six buildings for an average of 13.33 months participated in a study that assessed color preference (hue and lightness), lightness preference, and the effects of color on studying and mood. The results showed a preference for blue interiors, followed by green, violet, orange, yellow, and red. A preference bias was found for the specific color in which the student lived. Gender differences emerged for the preference of blue and violet. Room-lightness was significantly affected by the interior color. Room ceiling was preferred white. Blue as interior color was considered to facilitate studying activity. The use of differentiated colors in the six buildings was evaluated to significantly facilitate orienting and wayfinding. A significant relation was found between a calm mood and preference for blue.

## Introduction

Although color is a ubiquitous property of every architectural surface, evidence-based research on chromatic preference in architecture and psychological effects of color as a function of the architectural design of a space is still sparse. This study exploited a unique architectural setting of a university residence hall for long-term student accommodation, composed by six separate buildings that matched for every design feature with the only exception of interior color. Each building interior was characterized by a specific color for walls, ceiling, and floor in both common spaces and students’ rooms. The colors were: violet, blue, green, yellow, orange, and red. Students were independently assigned to the different colors by the residence administration. Therefore, this architectural setting resulted as an *in vivo* experiment that allowed a controlled assessment of students’ color preferences, their satisfaction with the color and lightness level of the building they lived in, and their assessment of the building color effect on their mood, studying activity, orienting within the residence.

Colors have three basic perceptual attributes: hue, saturation, and lightness (Hunt and Pointer, 2011). Hue is the phenomenological correspondent of wavelength within the visible-light spectrum. Saturation (chroma) describes the intensity or purity of a hue, whereas lightness (value) varies according to the relative presence of black or white in the color. At the lower extreme of saturation lie the achromatic colors gray, black, and white. Many models exist that map colors along these attributes in two-dimensional or three-dimensional spaces. Some of these models are perceptually uniform, and match human-color perception (e.g., Munsell, CIE Lab), whereas others are not perceptually uniform, and were developed to map colors for specific technical domains (e.g., RGB, HSV, HSL, HSB, CMYK).

Color preferences were mainly investigated manipulating hue, starting from the pioneering work by Eysenck (1941) who established a universal preference hierarchy in colors. According to his study the most preferred color was blue, followed by red, green, violet, orange, and yellow. This finding agreed with those obtained by Granger (1952) and Guilford and Smith (1959) who found the highest preference ratings for the blue-green hues and the lowest for yellow and yellow-green hues. These results were further confirmed by Granger (1955), Dittmar (2001), Bakker et al. (2013), and Schloss et al. (2013). Hue preferences in adults follows a relatively smooth curvilinear function in which cool colors (green, cyan, blue) are generally preferred to warm colors (red, orange, yellow) (Palmer et al., 2013). Focusing on color saturation, Palmer et al. (2013), in a review on color preference studies, concluded that, in general, more colorful and saturated colors are preferred to less vivid color. Saturation interacts with preferences for lightness so that yellow is preferred at high lightness levels, red and green at medium lightness levels, and blue and purple at low lightness levels (Guilford and Smith, 1959). Dark shades of orange (browns) and yellow (olives) tend to be strongly disliked relative to lighter, equally saturated oranges and yellow (Guilford and Smith, 1959; Palmer and Schloss, 2010). Color preference in these studies was assessed rating preselected color patches (either as physical colored chips, or presented on computer monitor), or asking participants to imagine colors, and was not referred to specific objects.

The extent to which these global and abstract color preferences could be applied to specific contexts was the focus of different studies. For example, Taft (1997) compared the abstract semantic ratings of color samples with those of the same colors applied to a variety of familiar objects (e.g., sofa, modern chair, antique chair, bicycle, cheese slicer, and computer), finding a good correspondence between the two sets of ratings. Overall, he found that only in the 4% of cases the color on the sample was judged different for attractiveness from the same color on an object. The specificity of color preference for specific objects was explained in terms of appropriateness of the color-object association based on people experience. Some objects, in fact, can be found in a wide variety of colors (e.g., bicycles), whereas many objects appear in a very limited range of colors (e.g., computers, smartphones). Schloss et al. (2013) showed eight hues, each at two levels of saturation and two levels of lightness, in addition to five achromatic colors (black, white, and three shades of gray). Participants had to rate the preference of each color contextless on simple patches, and with reference to different objects, both imagined and depicted (e.g., car, t-shirt, walls, sofa etc.). The results showed that people preferred more saturated colors when evaluating simple patches than real objects. They also preferred darker colors for objects (e.g., t-shirts, scarfs, and couches) compared to participants’ general preferences, with the exception of walls that were preferred with lighter colors. Furthermore, wall colors were preferred lighter in the imagined condition compared to the depicted condition. Jonauskaite et al. (2016) investigated context-specific color preferences comparing abstract color preferences, imagined interior walls, and imagined t-shirts. They used an unrestricted color selection approach with three-color dimensions (i.e., hue, chroma and lightness). Abstract colors were preferred with more chroma, whereas lighter colors were preferred for walls, and darker colors were preferred for t-shirts.

In the specific architectural context, Kunishima and Yanase (1985) investigated the visual effects of wall colors in living rooms. Architectural students had to evaluate living room models differing in color. A factor analysis highlighted three main dimensions: “activity,” “evaluation,” and “warmness.” “Activity” was mostly affected by the brightness of the wall color, “evaluation” by the saturation, and “warmness” by the hue.

The impact of light and color on psychological mood in work environments was investigated by Küller et al. (2006) in a large-scale study that involved 988 persons from different countries. The presence of some colors, in comparison to a no-color, or neutral-color condition, resulted in a more positive worker’s mood. The use of very saturated colors, to the contrary, had a negative effect on mood.

Several studies investigated the role of sex and culture to test the universality of color preference (Choungourian, 1968; Saito, 1994, 1996; Ou et al., 2004, 2012; Hurlbert and Ling, 2007; Al-Rasheed, 2015). A study on sex differences found a peak for the blue-green in the preference pattern of males and a peak for the reddish-purple region for females but when Chinese and British participants were analyzed separately the sex differences emerged only in the British subpopulation (Hurlbert and Ling, 2007).

Taylor et al. (2013a) pointed out that previous studies focused mainly on industrialized cultures, and they decided to compare color preferences of British adults to those of Himba adults, who belong to a non-industrialized culture in rural Namibia. Results suggested that predictive models proposed in previous studies cannot account for the differences observed in the two populations.

Another cross-cultural study found significant differences between a population from Poland and a population from Papua (Sorokowski et al., 2014), even if sex patterns had a much higher effect size than cultural difference. In fact, although preferences observed in the two populations were different, the differences observed in the preference patterns of males and females were comparable in the two samples.

Some studies also investigated the relation between color preferences and age (Teller et al., 2004; Zemach et al., 2007; Franklin et al., 2008, 2010; Taylor et al., 2013b). For hue preference there is good agreement between different age categories. In particular, both infants and adults tend to show a preference for blue and a dislike for greenish-yellow (Teller et al., 2004; Zemach et al., 2007; Franklin et al., 2008, 2010). Palmer and Schloss (2010) found significant differences for lightness and saturation (infants tend to prefer saturated and light hues). In comparative studies, the preference for blue was also confirmed in rhesus monkeys (Humphrey, 1972; Sahgal et al., 1975), and pigeons (Sahgal and Iversen, 1975).

Different accounts have been proposed to explain color preference. According to Hurlbert and Ling (2007) color preference is rooted in the cone-opponent contrast neural mechanisms which encode colors. Human color vision is in fact based on two cone-opponent systems, loosely called “red-green” and “blue-yellow.” The red-green system responds to the difference between long-wavelength-sensitive cone responses (L) and middle-wavelength-sensitive (M) responses (L–M), while the blue-yellow system differences short-wavelength-sensitive (S) cones with a combination of L and M cones [S – (L + M)]. The blue-yellow system accounts for the greatest variance (44.5%) for color preference across the population, with blue hues that are preferred over yellow hues. To the contrary, the red-green system accounts mainly for sex differences, with females that prefer colors with “reddish” contrast against the background in comparison to males (Hurlbert and Ling, 2007).

In another perspective, color preference could be grounded on emotional associations of colors. Colors are strictly associated to specific emotional states (Ou et al., 2004), and if an emotional state is perceived as pleasant then indirectly the pleasantness is transferred to the color. According to this theory, active, light, and cool colors are being preferred over passive, heavy, and warm ones. This theory, however, fails to explain why although blue is associated with sadness it is the most preferred color, and why yellow which is associated with joy, is less preferred than blue.

According to the ecological valence theory (EVT, Palmer and Schloss, 2010) color preferences arise from people’s average affective responses to color-associated objects, so that people like colors strongly associated with objects they like and dislike colors strongly associated with objects they dislike. For example, since water is important for surviving and water tends to be blue, blue is largely appreciated; similarly, since rotten food is dangerous for our health and rotten food tend to be greenish-yellow, this color is largely unappreciated. The EVT is able to explain both the universal trends and the minor variations: blue is probably appreciated in every culture while red is generally less appreciated, but for example the lucky effect that Chinese culture associate to this color make it more appreciable in China compared to other countries. The authors of the EVT estimated that the affective valence association was able to account for the 80% of variance in color preference ratings over 32 different colors.

Few controlled studies have investigated psychological and physiological effects of specific color exposure. For example, Jacobs and Hustmyer (1974) measured the physiological activation during a 1-min exposure to four different colors. Considering the galvanic skin response, red was significantly more arousing than other colors. Küller et al. (2009) compared psychological and physiological effects of a gray, red, and blue room. The results showed that the red room increased the brain arousal level (assessed as percentage of alpha waves). This effect was particularly significant in introvert persons or persons that were in a negative mood. Red was also found to be associated with a higher probability of winning a sport competition (Hill and Barton, 2005), to performance impairment on achievement tasks due to avoidance motivation (Elliot et al., 2007; Mehta and Zhu, 2009), and to performance enhancement on detail-oriented tasks (Mehta and Zhu, 2009).

Kwallek et al. (1996) compared nine monochromatic office interior colors in a between-subjects study in which university students performed a proofreading task in one office for a total permanence of 45 min. The nine office colors varied for two levels of saturation (high/low), and two levels of lightness (dark/light). Pre and post mood change and color preferences were also recorded. The proofreading task performance was not affected by office color, whereas errors were higher in the white office in comparison to the blue and red offices, even if it cannot be excluded that this difference could stem from cognitive differences between the groups in the different conditions. Higher saturated color offices resulted in higher vigor scores for mood. Lightness and coolness or warmth of the office color did not influence mood. Pleasantness for the office color differed significantly between the groups. Individuals preferred to work in beige and white rooms than in orange and purple offices. In terms of whether they liked the office color, individuals in the green and red offices preferred their office color more than individuals in the yellow and orange offices. Participants in the white, beige, blue, and gray offices liked the color of their offices more than participants in the orange office. Concerning the distracting effect of the color, participants in the purple, orange, red, yellow office colors reported that their colors were more distracting compared to participants in the green, gray, beige, and white offices. Purple and yellow office colors were rated as the most distracting, and white as the least distracting.

In the context of criminal detention holding cells Pellegrini et al. (1981) found no difference in the incidence of aggressive officer-arrestee encounters after changing the cell color from pale blue to hot pink.

Independently from the influence of color on behavior, people strongly tend to associate colors to specific semantic clusters (Sutton and Altarriba, 2016). Bright colors (e.g., white, pink) are often associated to positive emotions whereas dark colors (e.g., black, brown) tend to be associated with negative emotions (Hemphill, 1996). Furthermore, we tend to infer the valence of a stimulus on the basis of brightness (Meier et al., 2004). Individuals were faster to categorize positive words when they appeared in white than when they appeared in black, with an opposite trend for negative words. Color associations are often cross-modal (Spence, 2011), and the most important cross-modal association is the distinction between cold and warm colors (Ho et al., 2014).

Most of the literature that we have so far reviewed defined color effects and preferences exposing participants to colors via computer screens or using colored patches, or asking participants to imagine specific colors; furthermore, the exposure time to colored settings was in general very short (Elliot and Maier, 2014). The more realistic setting in an architectural study was that reported by Kwallek et al. (1996), but also in this case participants remained in the experimental room only the time to complete some tests for a total duration of about 45 min.

This is the first study that examined color preferences and the effects of environmental color on psychological functioning in a population that lived in mean more than 1 year in an architectural setting characterized by a strong monochromatic color interior design. The innovative aspect of our study was the possibility to examine color preferences and psychological effects of long-term color exposure in a real residential context. The university residence hall provided a setting with a high ecological validity for the study of color influence on residential satisfaction, lightness self-evaluation, study facilitation, and mood.

## Materials and Methods

### Participants

Participants were 443 university students living in a university residence hall. The sample included 230 males (*M_ag_*_e_ = 23.91, *SD* = 2.73) and 213 females (*M_ag_*_e_ = 23.68, *SD* = 2.60). The distribution of participants between the six buildings, differing for the specific interior color, was: orange *N* = 74 (16.7%), blue *N* = 75 (16.9%), yellow *N* = 74 (16.7%), red *N* = 87 (19.6%), green *N* = 85 (19.2%), and violet *N* = 48 (10.9%). Student assignment to the different buildings was performed by the residence hall administration at the time of admission. Mean stay at the university residence hall at the time of the research was 13.33 months (*SD* = 12.14). Difference between mean stay in the six buildings was not significant.

Participants were accommodated in single (31.8%) and double (68.2%) rooms. The proportion of students in single and double rooms was homogeneous for the six buildings. Eight participants were excluded because they declared a deficiency in color vision. Participants declared to spend an average of 6.78 h (*SD* = 3.28) per day in their room (excluding sleeping time).

This study was carried out in accordance with the recommendations of the Ethics Committee of the University of Bologna that approved the study protocol. All participants gave written informed consent in accordance with the Declaration of Helsinki. The data were collected in an anonymous form.

### Procedure and Data Analysis

**FIGURE 1:**
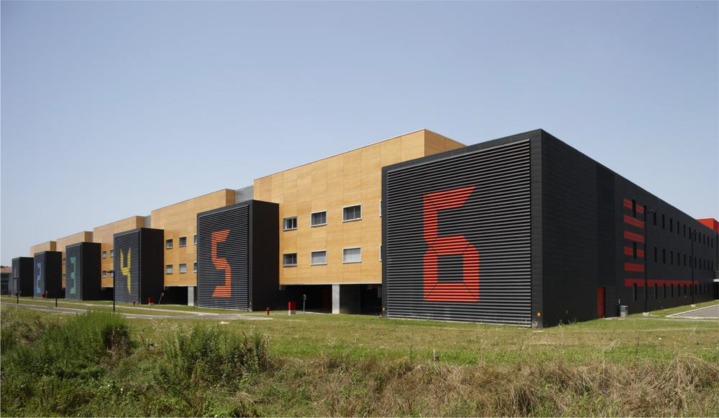
The six buildings of the “I Praticelli” university residence hall with number and color assignment.

The study was conducted at the university residence hall “I Praticelli,” located in Pisa (Italy). This setting was chosen for its architectural properties, since the university residence hall is divided into six identical buildings differing only for the interior color (walls, floor, and ceiling) (**Figure 1**). Each building has common areas (corridors, kitchen, and living roy coordinates are reported in **Table 1**. Artificial lighting within the six buildings was uniform with the use of linear fluorescent bulbs (photometrical data: color rendering index Ra ≥ 80, light color 830, rated color temperature 3000K).

**Table 1:** RAL code, color sample, and CIE Yxy coordinates for walls, ceilings, and floors of the six buildings considered in the study.

Building	Walls and ceiling			Floor		
	RAL	Sample	CIE Yxy	RAL	Sample	CIE Yxy
Red	3020	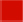	11.531, 0.6157, 0.3373	3016	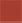	11.650, 0.5337, 0.3472
Orange	2009	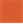	21.731, 0.5771, 0.3771	3012	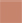	29.612, 0.4168, 0.3599
Yellow	1023	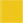	52.822, 0.4772, 0.4593	1000	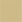	49.875, 0.3701, 0.3912
Green	6018	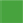	25.640, 0.3359, 0.5034	6021	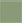	29.518, 0.3293, 0.3856
Blue	5012	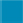	21.269, 0.2020, 0.2470	5024	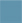	26.333, 0.2469, 0.2864
Violet	4009	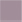	26.294, 0.3260, 0.3103	4009	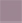	26.294, 0.3260, 0.3103

**FIGURE 2:**
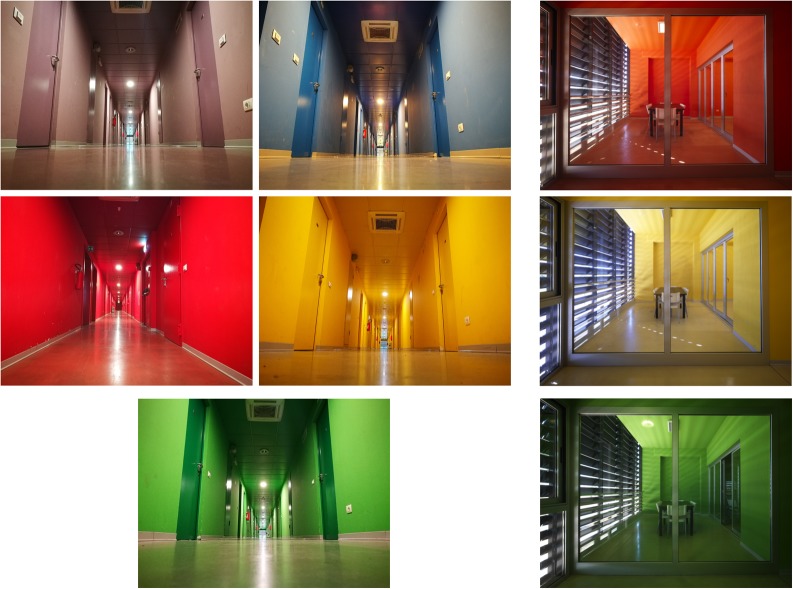
Examples of five corridors **(left)** and three study rooms **(right)** of the university residence hall.

**FIGURE 3:**
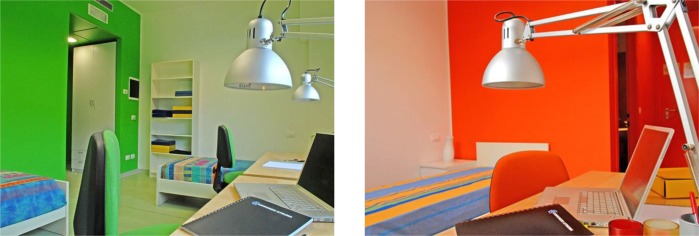
Example of a green and orange student’s room at the university residence hall.

The layout of students’ rooms included a small entrance hall connected to the bathroom and the main room (**Figure 3**). The specific color that characterized each building was applied to: (a) walls and ceiling of all interior common spaces (i.e., corridors, kitchens, study rooms); (b) walls and ceiling of the entrance hall inside the student’s room; (c) one wall in the bedroom (the remaining walls and the ceiling in the bedroom were painted in white); (d) walls of the bathroom (the ceiling was white) (**Figures 2**, **3**). Floors, both inside the room and in the common spaces, had a color coherent with the building but slightly different in hue from the wall colors. Floor-color coordinates and RAL codes for the six buildings are reported in **Table 1**.

**FIGURE 4:**
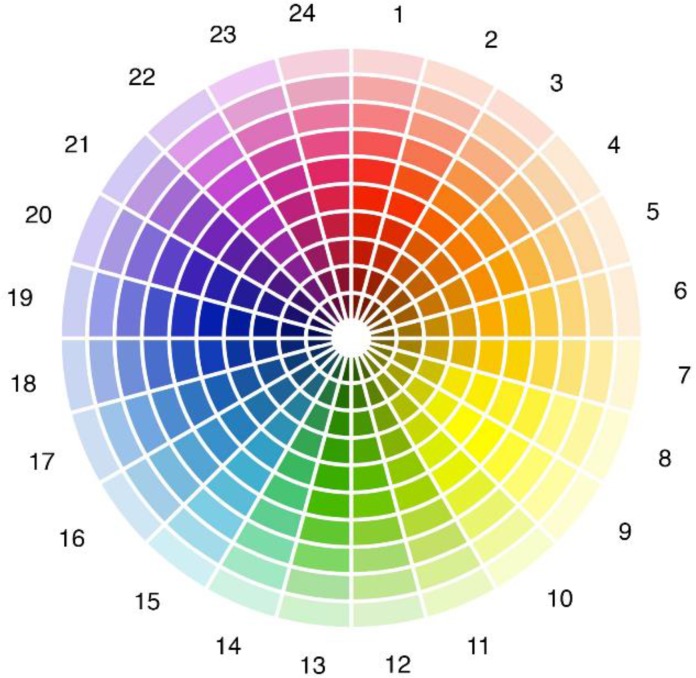
Color wheel for the assessment of chromatic preference. The wheel included 24 sectors varying in hue. Each sector included 10 levels along the radial dimension varying in lightness.

Color preferences and the student’s experience with the university hall design were investigated administering a questionnaire structured in these sections: (a) socio-demographic data (age, sex, province of residence), and university course attended by the student; (b) color vision deficiency; (c) hall of residence color in which the student lived (red, orange, yellow, green, blue, violet); (d) hall of residence color in which the student would prefer to live; (e) time stayed at the residence hall since admission; (f) room type (single, double); (g) color lightness preference for the building in which the student lived; (h) color preference in general (**Figure 4** considering both hue and lightness); (i) color preference for the residence hall (**Figure 4**, both hue and lightness); (j) room ceiling preference (white, colored); (k) room lightness level (-2/+2); (l) room lightness satisfaction (-2/+2); (m) hours per day spent in the room; (n) facilitating effect of the specific building color in the studying activity (-2/+2); (o) effect of the hall of residence color scheme for wayfinding and orienting (0–3).

When evaluating general color preference and room color preference the student had to choose a specific sample from the color wheel shown in **Figure 4**. The color wheel was divided into 24 sectors differing in hue. Each sector was divided in 10 levels differing in lightness along the radial dimension, for a total of 240 color samples. For each sample we considered the CIE Yxy coordinates, and Y values were considered as a proxy of lightness level in the range 0–100.

In addition to the questionnaire, the students were administered the *Brief Mood Introspection Scale* (Mayer and Gaschke, 1988) for an assessment of general mood. This scale assesses mood along these dimensions: happy, loving, calm, energetic, fearful/anxious, angry, tired, and sad. Each dimension includes two items for a total of 16 ratings: lively, happy, sad, tired, caring, content, gloomy, jittery, drowsy, grouchy, peppy, nervous, calm, loving, fed up, and active.

## Results

### Interior Color Preference

Blue was the preferred interior color (34.7%), followed by green (23.1%), violet (14.1%), orange (11.9%), yellow (8.7%), and red (7.5%). The Chi-square that tested non-equality in frequency distribution was significant: χ^2^ = 116.52, *p* < 0.001, φ = 0.55.

**FIGURE 5:**
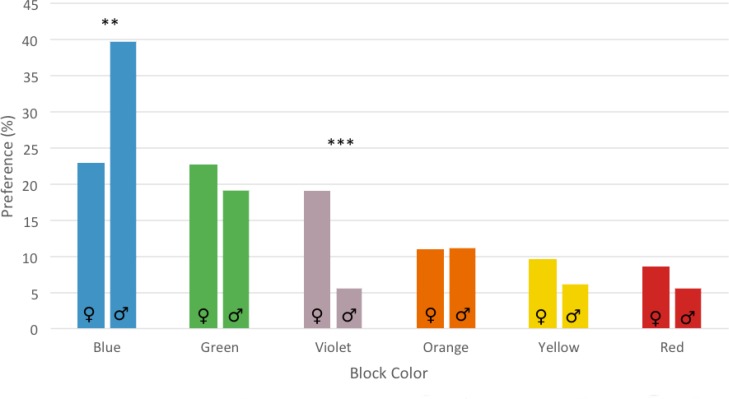
Interior color preference for the six buildings in male and female participants. Asterisks show the significance level of the gender difference test. ^∗∗^*p* < 0.01, ^∗∗∗^*p* < 0.001.

Interior color preference as a function of participant’s sex is shown in **Figure 5**. Separate Chi-square analysis with Bonferroni correction were performed to test the effect of sex on each interior color preference. The difference was significant for blue (χ^2^ = 6.03, *p* = 0.01), and violet (χ^2^ = 18.13, *p* < 0.001), as shown in **Figure 5**.

Color preference as a function of participant’s residence hall color is shown in **Table 2**. The frequency cross-tabulation analysis was significant: χ^2^ = 364, *p* < 0.001, φ = 0.89. Superimposed to the general preference for the blue and green hall of residences, participants showed a preference bias for the color in which they actually lived (diagonal entries in **Table 2**). For example, although red was the least preferred interior color with a mean choice of 7.5%, 28% of the participants living in the red hall of residence preferred to stay in that color. Similarly, yellow was preferred only by 8.7% of participants, but 31.7% of the students living in the yellow hall of residence preferred that specific color. The effects were summed for the most preferred interior colors: 53.6% of the students living in the blue hall of residence preferred not to change, and 48.6% of those living in the green rooms preferred to stay in that specific hall of residence.

**Table 2:** Residence color preference (%) as a function of the actual residence color in which the student lived.

		Desired hall of residence color					
**Participant’s actual hall of residence color**		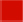	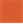	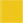	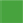	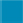	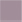
	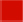	28	9.3	4	22.7	28	8
	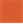	1.6	42.2	4.7	12.5	28.1	10.9
	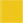	0	6.7	31.7	23.3	28.3	10
	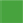	6.8	5.4	2.7	48.6	28.4	8.1
	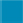	2.9	2.9	5.8	14.5	53.6	20.3
	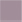	2.3	7	7	11.6	37.2	34.9
	Mean	7.5	11.9	8.7	23.1	34.7	14.1

Interior color preference was not affected by the type of accommodation (i.e., single *versus* double).

### General Chromatic Preference

Participants had to select the preferred color between the 240 samples included in **Figure 4**. The preference was general and not referred to a specific object or context. Hue (wheel sector) and lightness (radial axis) were separately analyzed.

Grouping the 24 hues into six main categories, color preference in descending order was: blue (39.2%), green (18.8%), red (18.6%), violet (9.3%), orange (8.4%), and yellow (5.7%). These preferences were significantly affected by the specific color in which the participant lived: *t*(5) = -4.71, *p* = 0.005, ${\text{η}}_{\text{p}}^{2}$ = 0.81. The bias was in mean +5.42% (*SD* = 3.08%) in favor of the color to which the student belonged, and ranged from 1.19% for students of the red hall of residence to 9.60% for students living in the blue hall of residence. The bias was computed with the difference between mean preference for a specific color for all participants and mean preference for a specific color considering only those that lived in that color. Accommodation in a single *versus* double room was not critical for general chromatic preference.

**FIGURE 6:**
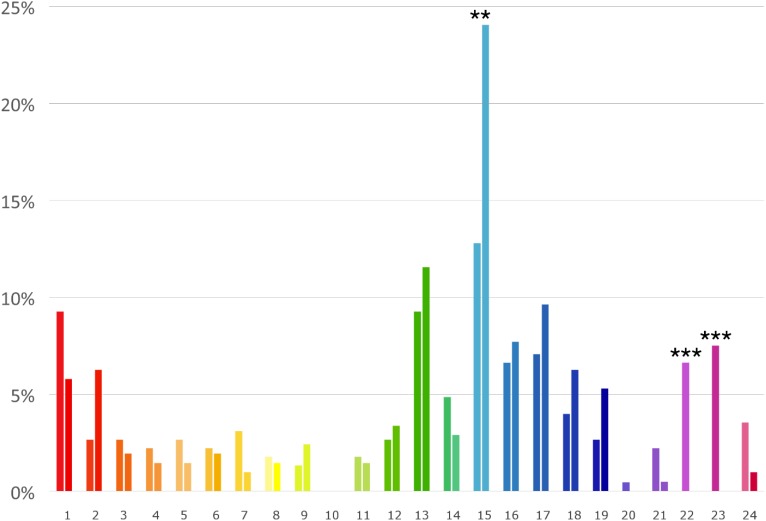
General chromatic preference (%) in females (left bar) and males (right bar) for the 24 hues shown in **Figure 4**. Lightness level of each bar matches the mean preferred lightness level (radial axis in **Figure 4**). Asterisks show the significance level for the male-female comparison (^∗∗^*p* < 0.01, ^∗∗∗^*p* < 0.001).

Preference for each of the 24 hues, distinguishing between male and female participants, is shown in **Figure 6**. Male and female preferences were significantly different for hue 15 (χ^2^ = 5.58, *p* = 0.001), hue 22 (χ^2^ = 15, *p* < 0.001), and hue 23 (χ^2^ = 17, *p* < 0.001) (**Figure 6**).

**FIGURE 7:**
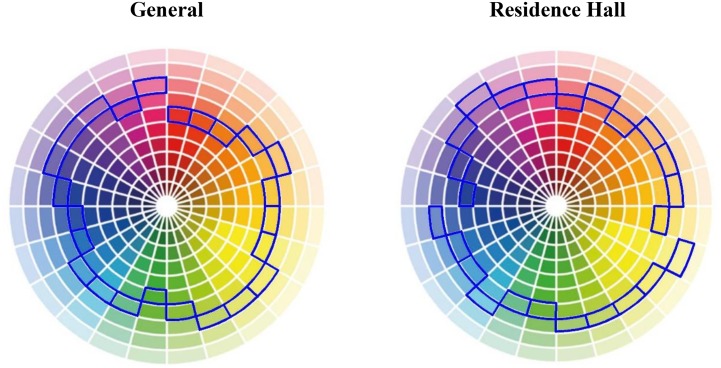
Preferred lightness level (blue frame), for each of the 24 hues, in the general condition **(left)** and in the university residence hall condition **(right)**.

Lightness preference was tested with an ANOVA inserting hue (24 levels) and sex as factors. Main effect for hue was significant *F*(22,408) = 27,46, *p* < 0.001, ${\text{η}}_{\text{p}}^{2}$ = 0.59. The preferred lightness level for each hue is shown in **Figure 7** (left). Sex and the interaction between hue and sex were not significant.

### Residence Hall Chromatic Preference

Grouping the 24 hues of **Figure 4** into six main categories, specific color preferences for the university residence hall were: blue (36.4%), green (20.8%), orange (12.5%), yellow (11.2%), red (10.5%), and violet (8.6%). These preferences were significantly influenced by the color in which the participant lived: *t*(5) = -4.99, *p* = 0.004, ${\text{η}}_{\text{p}}^{2}$ = 0.83. The preference bias for the own color was in mean 9.14% (*SD* = 4.48%), and ranged from 3.74% for the students living in the yellow hall to 13.70% for those living in the blue hall. The bias was computed subtracting mean preference for a specific color considering all participants to mean preference for a specific color including only those living in that color. The type of accommodation (single *versus* double) was not critical.

**FIGURE 8:**
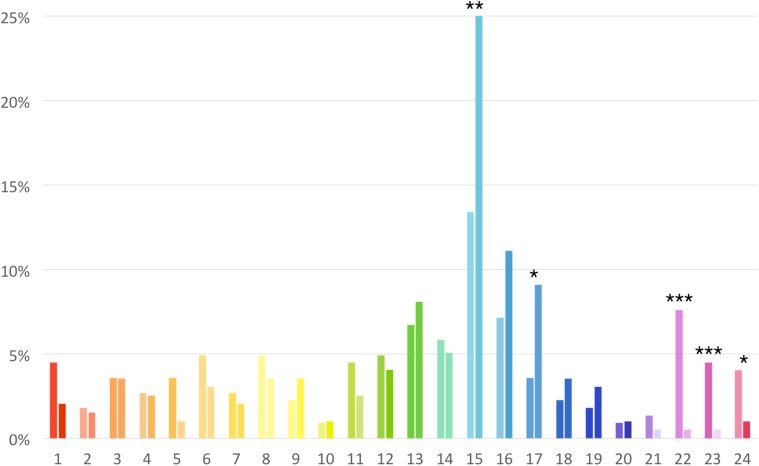
Distribution of the residence hall color preference for males **(right bars)** and females **(left bars)** for the 24 hues considered in the study. Lightness level of each bar matches the average preferred lightness level. Asterisks show the significance level for the male-female comparison (^∗^*p* < 0.05, ^∗∗^*p* < 0.01, ^∗∗∗^*p* < 0.001).

The distribution of preferences for each of the 24 hues in males and females is shown in **Figure 8**. Each bar color in **Figure 8** shows also the preferred lightness level for each hue. Chromatic preference for male and female participants differed significantly in five hues: hue 15 (χ^2^ = 5.90, *p* = 0.01), hue 17 (χ^2^ = 3.84, *p* = 0.04), hue 22 (χ^2^ = 14.22, *p* < 0.001), hue 23 (χ^2^ = 7.36, *p* = 0.006), and hue 24 (χ^2^ = 4.45, *p* = 0.03).

Color lightness preference for the residence hall was tested with an ANOVA inserting hue (24 levels) and sex as factors. Main effect for hue was significant *F*(23,393) = 24.46, *p* < 0.001, ${\text{η}}_{\text{p}}^{2}$ = 0.59. The preferred color lightness level for each hue is shown in **Figure 7** (right). Sex and the interaction between hue and sex were not significant.

### General Versus Residence Hall Color Lightness Preference

General color lightness preference was compared to the residence hall color lightness preference with a matched *t-*test including all the 24 hue sectors. The *t*-test was significant: *t*(23) = -2.58, *p* = 0.02, η^2^= 0.22. Mean preferred chromatic lightness level was 42.82 (*SD* = 24.23) in the general evaluation, and 46.76 (*SD* = 23.76) when referred specifically to the residence hall.

### Preferred Color Lightness Level

**FIGURE 9:**
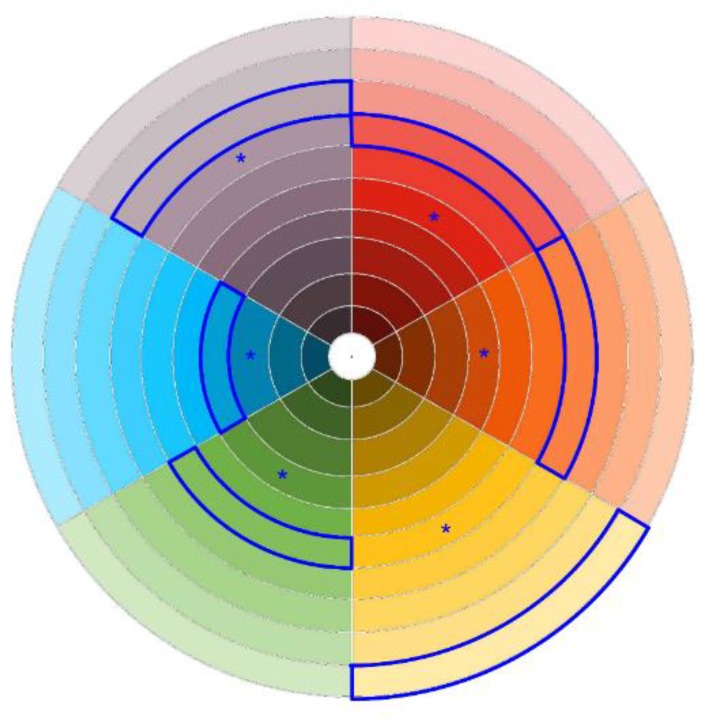
Color lightness preference (blue frame) as a function of the six-building interior color. The asterisks show the actual color lightness level in the six buildings.

Participants had first to select the hue sector matching their building on the color wheel in **Figure 9**, then they had to mark the preferred lightness level on the wheel radius. Lightness level was operationalized as the Y value of the Yxy CIE coordinates of the selected patch.

Preferred lightness level differed significantly as a function of hue: *F*(5,175) = 34.03, *p* < 0.001, ${\text{η}}_{\text{p}}^{2}$ = 0.49. Framed swatches in **Figure 9** show the preferred color lightness level as a function of the building color. Mean preferred lightness for the six colors were: *M_yellow_* = 80.59 (*SD* = 14.11), *M_violet_* = 46.45 (*SD* = 17.72), *M_green_* = 45.78 (*SD* = 17.14), *M_orange_* = 38.60 (*SD* = 13.88), *M_blu_* = 32.70 (*SD* = 21.97), *M_red_* = 29.13 (*SD* = 20.74). Planned comparisons showed that the yellow painting was preferred lighter than all other paintings (*p* < 0.001). Furthermore, violet painting was preferred lighter than red painting (*p* = 0.008).

Preferred color lightness was compared to the actual color lightness of the six interior colors with a paired *t*-test that was significant: *t*(186) = 14.24, *p* < 0.001, η^2^ = 0.52. Mean preferred color lightness was 45.89 (*SD* = 24.56), significantly higher than the mean actual interior color lightness of the six buildings: 26.03 (*SD* = 14.02) (**Figure 9**). Being the student in a single *versus* double room was not critical.

### Room-Lightness Level

Self-evaluated room-lightness as a function of the room color was tested with an ANOVA that was significant: *F*(5,437) = 4.25, *p* = 0.001, ${\text{η}}_{\text{p}}^{2}$ = 0.05. On a -2/+2 scale mean room-lightness ratings were: 1.04 (*SD* = 0.12) in the yellow building, 0.71 (*SD* = 0.12) in the orange building, 0.65 (*SD* = 0.11) in the green building, 0.62 (*SD* = 0.15) in the violet building, 0.51 (*SD* = 0.11) in the red building, and 0.29 (*SD* = 0.12) in the blue building. Planned comparisons showed these significant contrasts: yellow rooms were evaluated lighter than blue rooms (*p* < 0.001), red rooms (*p* = 0.007), and green rooms (*p* = 0.40). Blue rooms were evaluated darker than orange rooms (*p* = 0.006), green rooms (*p* = 0.02), and violet rooms (*p* = 0.002).

### Room-Lightness Satisfaction

Room-lightness satisfaction as a function of the room interior color was tested with an ANOVA that was significant: *F*(5,437) = 4.33, *p* = 0.001, ${\text{η}}_{\text{p}}^{2}$ = 0.05. On a +2/-2 scale mean room-lightness satisfaction was 0.95 (*SD* = 0.12) for yellow rooms, 0.85 (*SD* = 0.15) for violet rooms, 0.71 (*SD* = 0.12) for orange rooms, 0.62 (*SD* = 0.11) for green rooms, 0.52 (*SD* = 0.11) for red rooms, and 0.25 (*SD* = 0.12) for blue rooms. Planned comparisons showed these significant contrasts: orange *versus* blue (*p* = 0.01), yellow *versus* blue (*p* < 0.001), green *versus* blue (*p* = 0.03), yellow *versus* red (*p* = 0.001), yellow *versus* green (*p* = 0.02). The correlation between self-evaluated room-lightness level and room-lightness satisfaction was 0.78 (*p* < 0.001). Room lightness satisfaction was not affected by the student’s accommodation (single *versus* double).

### White *Versus* Colored Ceiling

A white ceiling (84.93%) was preferred more than a colored ceiling (15.07%): χ^2^ = 97.09, *p* < 0.001, φ = 0.44.

### Spatial Orientation and Wayfinding

The use of specific colors for the six buildings was evaluated to facilitate spatial orientation and wayfinding within the university residence hall: *M* = 2.06 (*SD* = 0.98) on a 0 -3 scale. The rating was not significantly affected by building color.

### Mood and Interior Color

The effect of building interior color on the *Brief Mood Introspection Scale* was tested with a MANOVA that was not significant.

### Mood and Color Preference

The effect of mood on color preference (coded on six levels) was tested with a MANOVA that was significant for the “calm” scale: *F*(6,400) = 2.64, *p* = 0.01, ${\text{η}}_{\text{p}}^{2}$ = 0.04. Mean ratings as a function of the interior color were: green 1.54 (*SD* = 1.05), blue 1.21 (*SD* = 1.65), violet 1.12 (*SD* = 2.01), red 1.04 (*SD* = 1.68), yellow 0.89 (*SD* = 1.53), and orange 0.61 (*SD* = 1.73). Blue *versus* orange (*p* = 0.02), green *versus* yellow (*p* = 0.04), green *versus* orange (*p* = 0.001) were the significant pairwise comparisons.

### Building Interior Color and Facilitation of Studying Activity

An ANOVA tested the interaction between building interior color and facilitation of the studying activity (-2/+2 scale) of the participants. The interaction was significant: *F*(5,434) = 2.44, *p* = 0.03, ${\text{η}}_{\text{p}}^{2}$ = 0.03. Mean ratings as a function of the interior color were: blue 0.34 (*SD* = 0.08), violet 0.19 (*SD* = 0.10), green 0.17 (*SD* = 0.07), yellow 0.13 (*SD* = 0.08), orange 0.01 (*SD* = 0.08), and red 0.01 (*SD* = 0.07). Pairwise comparisons showed that the significance was explained by the contrasts blue *versus* orange (*p* = 0.004) and blue *versus* red (*p* = 0.003).

## Discussion

Whereas color on external façades influences the perception of the overall urban design and has mainly an aesthetic role (Mougthtin et al., 1995), color in interior design could significantly affect residential satisfaction and psychological and social functioning in addition to having an aesthetic value. Color in interior design can be more easily personalized, strongly interacts with the color of other decorating objects, and its pleasantness could affect home attachment. In the specificity of our study, we exploited a unique architectural setting composed by six buildings that differed only for the interior color, investigating pleasantness for each specific color; how this pleasantness related to general chromatic preference, the effects of the interior color on lightness level and lightness satisfaction, and the effect of the color on the residents’ functioning and mood. This is the first study that examined the effects of interior colors in occupants who “lived in a specific color” for an average time of more than one year, filling a void in the color preference literature that has always focused on the effects of brief exposures to specific colors (Palmer et al., 2013). Interior colors, to the contrary, tend to shape the “domestic landscape” for long-term intervals, and is therefore important to study color preferences and color effects on a large-time scale, as in this study.

The building with blue interior color was the most preferred, followed by the green, violet, orange, yellow, and red building. Blue was also the preferred color when performing general chromatic preferences, consistently with previous literature (Eysenck, 1941; Granger, 1952; Guilford and Smith, 1959; Hurlbert and Ling, 2007; Palmer and Schloss, 2010). Considering all the six buildings, cool colors (blue, violet, and green) were preferred to warm colors (yellow, orange, and red). This pattern of preferences could be linked to the ecological valence theory (Palmer and Schloss, 2010) that posit a causal link between the preference for a color and the preference for objects that are characterized by that specific color. In this perspective the preference for blues and cyans could emerge as a consequence for the preference of clear sky and clean water, or for the association of blue with serenity and calm (Ou et al., 2004), qualities that probably are sought by students for their residential space.

Superimposed to the preference for specific colors of the university residence hall we found an effect of “color attachment” in which a significant part of students expressed a preference for the specific color in which they lived. Different elements could concur to explain this effect. A mere exposure effect (Bornstein, 1989), due to the familiarity with the actual color, could have contributed to increase its pleasantness. An additional cause could be the residential attachment that the student developed with the hall of residence in which he/she lived (Tognoli, 2003; Rioux et al., 2017).

In general we found a considerable overlap between general chromatic preferences and interior color preferences, with the only exception of lightness: colors in interiors were preferred lighter than when expressing general preferences. For each of the six colors used in the university residence hall the participants expressed a preference for a lighter version. The discrepancy was maximum for yellow, intermediate for red and orange, and small for green, blue, and violet. Interestingly yellow was preferred at high levels of lightness, whilst blue was preferred more dark. In general, the interior colors used in the university residence hall were evaluated too dark and saturated, and not fully adequate to a residential setting.

Although blue was the preferred interior color for both males and females, the polarization for blue was less pronounced in female participants than in males. Females, for example, expressed a discrete preference for the violet color that most males rejected. Gender differences emerged also in the general chromatic preferences, with a lower polarization for the blue color, and a higher preference for red, pink, and violet in females. These results are consistent with the tendency reported by Hurlbert and Ling (2007) who compared a British and a Chinese sample, and by Al-Rasheed (2015) who compared Arabic and English participants, and recently by Bonnardel et al. (2018) who compared gender differences in color preference among British and Indian students, finding in females a more distributed pattern of color preference, and a secondary, superimposed preference for pink-purple colors. However, although these gender differences, it should be emphasized that for both males and females blue was the most preferred color.

The interior color influenced significantly room-lightness level and room-lightness satisfaction. Interestingly, the colors associated with high lightness level and satisfaction were complementary to those associated to color preference. For example, blue interiors were the most preferred but also those that had the higher detrimental effect on lightness level and satisfaction, whereas yellow, which was among the most undesirable colors at the university residence, led to the highest levels of lightness satisfaction. This connection between interior color and lightness level is important considering that the amount of daylight in a residential or working environment is a predictor of stress reduction and satisfaction (Alimoglu and Donmez, 2005; Yildirim et al., 2007).

In general, there was a strong preference for rooms with a white ceiling, probably because the perceived room height increases with the ceiling lightness (Oberfeld et al., 2010).

The assignment of a specific color to each building was considered to facilitate orientation and wayfinding within the university residence hall, in line with the previous research of Hidayetoglu et al. (2012). Interior color is the primary source for increasing legibility and facilitating spatial navigation within a complex architecture. Furthermore, we can suggest that the characterization of each building with a specific color could have promoted a higher place attachment to the own building, as suggested also by the preference bias found for the color in which the student lived.

The blue interior color was considered to promote and facilitate studying activity more than lighter and warmer colors (such as orange and red) that probably were perceived as too arousing (Küller et al., 2009). Furthermore, we found an association between a blue color preference and the “calm” rating in the mood scale. These results can be explained considering that the color blue is often associated with openness, peace, and tranquility (Kaya and Epps, 2004), in contrast with red that is often associated with dangers, activation, erotic pleasure (Elliot et al., 2007). Furthermore, Mehta and Zhu (2009), from a series of six studies, demonstrated that blue (*versus* red), activated an approach motivation and enhanced performance on creative cognitive tasks. These results were further confirmed by Xia et al. (2016).

The interior colors that were evaluated to have the worst effect on studying were red and orange. This effect could be explained considering that long-wave colors can cause higher arousal than short-wave colors (Jacobs and Hustmyer, 1974; Walters et al., 1982). According to the Yerkes-Dodson law (Yerkes and Dodson, 1908), this high-arousal state could negatively affect the performance in difficult tasks, as studying.

As guidelines for the design of university residence halls and interiors in residential settings in general, we could suggest preferring blue and green colors and avoid red, yellow and orange colors. In case of a residence hall for male students it is better to restrict the color palette to only blue and green hues, whereas in case of female students the color palette could be more varied, including also red-purple and violet hues. The blue color is to be preferred in study areas. Light colors are to be preferred for preserving an adequate lightness level, and a white ceiling is preferred over a colored one. In general, when the university hall has a complex layout, segregating functional spaces with specific colors could be helpful for facilitating spatial orientation and wayfinding.

Interior color is a ubiquitous component of every architecture design that strongly characterizes residential, work, educational, commercial environments, and has a significant impact on psychological functioning and satisfaction on the people living in these environments. The development of applied research in this field could contribute to establish an evidence-based knowledge that can be used by designers and architects to guide color choice in their projects.

## Author Contributions

MC and SF designed and devised the study. SF acquired the data. MC, SF, and IP analyzed the data. MC, SF, MN, and IP wrote the manuscript.

## Conflict of Interest Statement

The authors declare that the research was conducted in the absence of any commercial or financial relationships that could be construed as a potential conflict of interest.

## References

[B1] Alimoglu M. K. Donmez L. (2005). Daylight exposure and the other predictors of burnout among nurses in a university hospital. *Int. J. Nurs. Stud.* 42 549–555. 10.1016/j.ijnurstu.2004.09.001 15921986

[B2] Al-Rasheed A. S. (2015). An experimental study of gender and cultural differences in hue preference. *Front. Psychol.* 6:30. 10.3389/fpsyg.2015.00030 25688219 PMC4311615

[B3] Bakker I. van der Voordt T. Vink P. de Boon J. Bazley C. (2013). Color preferences for different topics in connection to personal characteristic. *Color Res. Appl.* 40 62–71. 10.1002/col.21845

[B4] Bonnardel V. Beniwal S. Dubey N. Pande M. Bimler D. (2018). Gender difference in color preference across cultures: an archetypal pattern modulated by a female cultural stereotype. *Color Res. Appl.* 43 209–223. 10.1002/col.22188

[B5] Bornstein R. F. (1989). Exposure and affect: overview and meta-analysis of research. *Psychol. Bull.* 106 265–289. 10.1037/0033-2909.106.2.265

[B6] Choungourian A. (1968). Colour preferences and cultural variation. *Percept. Mot. Skills* 26 1203–1206. 10.2466/pms.1968.26.3c.1203 5675688

[B7] Dittmar M. (2001). Changing colour preferences with ageing: a comparative study on younger and older native Germans aged 19-90 years. *Gerontology* 47 219–226. 10.1159/000052802 11408728

[B8] Elliot A. J. Maier M. A. (2014). Color psychology: effects of perceiving color on psychological functioning in humans. *Annu. Rev. Psychol.* 65 95–120. 10.1146/annurev-psych-010213-115035 23808916

[B9] Elliot A. J. Maier M. A. Moller A. C. Friedman R. Meinhardt J. (2007). Color and psychological functioning: the effect of red on performance attainment. *J. Exp. Psychol. Gen.* 136 154–168. 10.1037/0096-3445.136.1.154 17324089

[B10] Eysenck H. J. (1941). A critical and experimental study of colour preferences. *Am. J. Psychol.* 54 385–394. 10.2307/1417683 28950922

[B11] Franklin A. Bevis L. Ling Y. Hurlbert A. (2010). Biological components of colour preference in infancy. *Dev. Sci.* 13 346–354. 10.1111/j.1467-7687.2009.00884.x 20136931

[B12] Franklin A. Drivonikou G. V. Bevis L. Davies I. R. L. Kay P. Regier T. (2008). Categorical perception of color is lateralized to the right hemisphere in infants, but to the left hemisphere in adults. *Proc. Natl. Acad. Sci.* 105 3221–3225. 10.1073/pnas.0712286105 18316729 PMC2265127

[B13] Granger G. W. (1952). Objectivity of colour preferences. *Nature* 170 778–780. 10.1038/170778a0 13002441

[B14] Granger G. W. (1955). An experimental study of colour preferences. *J. Gen. Psychol.* 52 3–20. 10.1080/00221309.1955.9918340

[B15] Guilford J. P. Smith P. C. (1959). A system of color-preferences. *Am. J. Psychol.* 72 487–502. 10.2307/1419491 13830144

[B16] Hemphill M. (1996). A note on adults’ color–emotion associations. *J. Gen. Psychol.* 157 275–280. 10.1080/00221325.1996.9914865 8756892

[B17] Hidayetoglu M. L. Yildirim K. Akalin A. (2012). The effects of color and light on indoor wayfinding and the evaluation of the perceived environment. *J. Environ. Psychol.* 32 50–58. 10.1016/j.jenvp.2011.09.001

[B18] Hill R. A. Barton R. A. (2005). Psychology: red enhances human performance in contests. *Nature* 435 293–293. 10.1038/435293a 15902246

[B19] Ho H.-N. Van Doorn G. H. Kawabe T. Watanabe J. Spence C. (2014). Colour-temperature correspondences: when reactions to thermal stimuli are influenced by colour. *PLoS One* 9:e91854. 10.1371/journal.pone.0091854 24618675 PMC3950287

[B20] Humphrey N. K. (1972). “Interest” and “pleasure”: two determinants of a monkey’s visual preferences. *Perception* 1 395–416. 10.1068/p010395 4680941

[B21] Hunt R. W. G. Pointer M. R. (2011). *Measuring Colour* 4th Edn. Chichester: Wiley 10.1002/9781119975595

[B22] Hurlbert A. C. Ling Y. (2007). Biological components of sex differences in color preference. *Curr. Biol.* 17 623–625. 10.1016/j.cub.2007.06.022 17714645

[B23] Jacobs K. W. Hustmyer F. E. (1974). Effects of four psychological primary colors on GSR, heart rate and respiration rate. *Percept. Mot. Skills* 38 763–766. 10.2466/pms.1974.38.3.763 4842431

[B24] Jonauskaite D. Mohr C. Antonietti J. P. Spiers P. M. Althaus B. Anil S. (2016). Most and least preferred colours differ according to object context: new insights from an unrestricted colour range. *PLoS One* 11:e0152194. 10.1371/journal.pone.0152194 27022909 PMC4811414

[B25] Kaya N. Epps H. H. (2004). Relationship between color and emotion: a study of college students. *Coll. Stud. J.* 38 396–405.

[B26] Küller R. Ballal S. Laike T. Mikellides B. Tonello G. (2006). The impact of light and colour on psychological mood: a cross-cultural study of indoor work environments. *Ergonomics* 49 1496–1507. 10.1080/00140130600858142 17050390

[B27] Küller R. Mikellides B. Janssens J. (2009). Color, arousal, and performance - A comparison of three experiments. *Color Res. Appl.* 34 141–152. 10.1002/col.20476

[B28] Kunishima M. Yanase T. (1985). Visual effects of wall colours in living rooms. *Ergonomics* 28 869–882. 10.1080/00140138508963208 4029112

[B29] Kwallek N. Lewis C. M. Lin-Hsiao J. W. D. Woodson H. (1996). Effects of nine monochromatic office interior colors on clerical tasks and worker mood. *Color Res. Appl.* 21 448–458. 10.1002/(SICI)1520-6378(199612)21:6<448::AID-COL7>3.0.CO;2-W

[B30] Mayer J. D. Gaschke Y. N. (1988). The experience and meta-experience of mood. *J. Pers. Soc. Psychol.* 55 102–111. 10.1037/0022-3514.55.1.102 3418484

[B31] Mehta R. Zhu R. J. (2009). Blue or red? Exploring the effect of color on cognitive task performances. *Science* 323 1226–1229. 10.1126/science.1169144 19197022

[B32] Meier B. P. Robinson M. D. Clore G. L. (2004). Why good guys wear white: automatic inferences about stimulus valence based on brightness. *Psychol. Sci.* 15 82–87. 10.1111/j.0963-7214.2004.01502002.x 14738513

[B33] Mougthtin C. Oc T. Tiesdell S. (1995). *Urban Design: Ornement and Decoration*. Oxford: Butterworth.

[B34] Oberfeld D. Hecht H. Gamer M. (2010). Surface lightness influences perceived room height. *Q. J. Exp. Psychol.* 63 1999–2011. 10.1080/17470211003646161 20401809

[B35] Ou L.-C. Luo M. R. Woodcock A. Wright A. (2004). A study of colour emotion and colour preference. Part I: colour emotions for single colours. *Color Res. Appl.* 29 232–240. 10.1002/col.20010

[B36] Ou L.-C. Ronnier Luo M. Sun P. L. Hu N. C. Chen H. S. Guan S. S. (2012). A cross-cultural comparison of colour emotion for two-colour combinations. *Color Res. Appl.* 37 23–43. 10.1002/col.20648

[B37] Palmer S. E. Schloss K. B. (2010). An ecological valence theory of human color preference. *Proc. Natl. Acad. Sci.* 107 8877–8882. 10.1073/pnas.0906172107 20421475 PMC2889342

[B38] Palmer S. E. Schloss K. B. Sammartino J. (2013). Visual aesthetics and human preference. *Annu. Rev. Psychol.* 64 77–107. 10.1146/annurev-psych-120710-100504 23020642

[B39] Pellegrini R. J. Schauss A. G. Miller M. E. (1981). Room color and aggression in a criminal detention holding cell: a test of the “tranquilizing pink” hypothesis. *J. Orthomol. Psychiatry* 10 174–181.

[B40] Rioux L. Scrima F. Werner C. M. (2017). Space appropriation and place attachment: university students create places. *J. Environ. Psychol.* 50 60–68. 10.1016/j.jenvp.2017.02.003

[B41] Sahgal A. Iversen S. (1975). Color preferences in the pigeon: a behavioral and psychopharmacological study. *Psychopharmacologia* 43 175–179. 10.1007/BF00421021 1187952

[B42] Sahgal A. Roderick Pratt S. Iversen S. (1975). Response preferences of monkey (*Macaca mulatta*) within wavelength and line-tilt dimensions. *J. Exp. Anal. Behav.* 24 377–381. 10.1901/jeab.1975.24-377 16811888 PMC1333439

[B43] Saito M. (1994). A cross-cultural study on color preference in three Asian cities. *Jpn. Psychol. Res.* 36 219–232. 10.4992/psycholres1954.36.219

[B44] Saito M. (1996). Comparative studies on color preference in Japan and other Asian regions, with special emphasis on the preference for white. *Color Res. Appl.* 21 35–49. 10.1002/(SICI)1520-6378(199602)21:1<35::AID-COL4>3.0.CO;2-6

[B45] Schloss K. B. Strauss E. D. Palmer S. E. (2013). Object color preferences. *Color Res. Appl.* 38 393–411. 10.1002/col.21756

[B46] Sorokowski P. Sorokowska A. Witzel C. (2014). Sex differences in color preferences transcend extreme differences in culture and ecology. *Psychon. Bull. Rev.* 21 1195–1201. 10.3758/s13423-014-0591-8 24570324 PMC4181517

[B47] Spence C. (2011). Crossmodal correspondences: a tutorial review. *Atten. Percept. Psychophys.* 73 971–995. 10.3758/s13414-010-0073-7 21264748

[B48] Sutton T. M. Altarriba J. (2016). Color associations to emotion and emotion-laden words: a collection of norms for stimulus construction and selection. *Behav. Res. Methods* 48 686–728. 10.3758/s13428-015-0598-8 25987304

[B49] Taft C. (1997). Color meaning and context: comparisons of semantic ratings of colors on samples and objects. *Color Res. Appl.* 22 40–50. 10.1002/(SICI)1520-6378(199702)22:1<40::AID-COL7>3.0.CO;2-4

[B50] Taylor C. Clifford A. Franklin A. (2013a). Color preferences are not universal. *J. Exp. Psychol. Gen.* 142 1015–1027. 10.1037/a0030273 23148465

[B51] Taylor C. Schloss K. Palmer S. E. Franklin A. (2013b). Color preferences in infants and adults are different. *Psychon. Bull. Rev.* 20 916–922. 10.3758/s13423-013-0411-6 23435629

[B52] Teller D. Y. Civan A. Bronson-Castain K. (2004). Infants’ spontaneous color preferences are not due to adult-like brightness variations. *Vis. Neurosci.* 21 397–401. 10.1017/S0952523804213360 15518220

[B53] Tognoli J. (2003). Leaving home: homesickness, place attachment, and transition among residential college students. *J. Coll. Stud. Psychother.* 18 35–48. 10.1300/J035v18n01_04

[B54] Walters J. Apter M. J. Svebak S. (1982). Color preference, arousal, and the theory of psychological reversals. *Mot. Emot.* 6 193–215. 10.1007/BF00992245

[B55] Xia T. Song L. Wang T. T. Tan L. Mo L. (2016). Exploring the effect of red and blue on cognitive task performances. *Front. Psychol.* 7:784. 10.3389/fpsyg.2016.00784 27303343 PMC4880552

[B56] Yerkes R. M. Dodson J. D. (1908). The relation of strength of stimulus to rapidity of habit formation. *J. Comp. Psychol.* 18 459–482. 10.1002/cne.920180503

[B57] Yildirim K. Akalin-Baskaya A. Celebi M. (2007). The effects of window proximity, partition height, and gender on perceptions of open-plan offices. *J. Environ. Psychol.* 27 154–165. 10.1016/j.jenvp.2007.01.004

[B58] Zemach I. Chang S. Teller D. Y. (2007). Infant color vision: prediction of infants’ spontaneous color preferences. *Vis. Res.* 47 1368–1381. 10.1016/j.visres.2006.09.024 17118421

